# Border tissue morphology is associated with macular ganglion cell thickness in open-angle glaucoma

**DOI:** 10.1038/s41598-022-26348-y

**Published:** 2022-12-19

**Authors:** Do Young Park, Yoon Kyung Jang, Ji Ho Kim, Jiyoun Choi, Wool Suh, Changwon Kee, Jong Chul Han

**Affiliations:** 1 Department of Ophthalmology, Yeungnam University Hospital, Yeungnam University College of Medicine, Daegu, Korea; 2 Department of Ophthalmology, Samsung Medical Center, Sungkyunkwan University School of Medicine, Seoul, Korea; 3 Department of Ophthalmology, Ewha Womans University Mokdong Hospital, Ewha Womans University College of Medicine, Seoul, Korea; 4 Department of Medical Device, Management and Research, SAIHST, Sungkyunkwan University, Seoul, Korea

**Keywords:** Optic nerve diseases, Glaucoma

## Abstract

Externally oblique border tissue (EOBT) configuration is topographically associated with glaucomatous damage in the optic nerve head. We investigated the relationship between the EOBT characteristics and macular retinal ganglion cell (RGC) thickness in patients with open-angle glaucoma (OAG). A total of 149 eyes with OAG that had an EOBT observed on optical coherence tomography exams were included. After determining the maximum EOBT length and angular location of the maximal EOBT length, we analyzed their correlation with macular ganglion cell inner plexiform layer (GCIPL) and peripapillary retinal nerve fiber layer (pRNFL) thickness. The macular GCIPL and pRNFL thickness were compared based on the angular location of the longest EOBT, and their association was assessed using multivariable regression analysis. Maximum EOBT length was significantly correlated with macular GCIPL thickness, but not with pRNFL thickness. Macular GCIPL was thinnest in eyes with EOBT located in a temporal direction to the optic disc. Longer maximum EOBT and temporally elongated EOBT were independently associated with a thinner macular GCIPL in the multivariable regression analysis. These suggest that temporal elongation of the EOBT may increase the stress and strain on the RGCs derived from the macula and make RGCs more susceptible to glaucoma-inducing damage.

## Introduction

Glaucoma is a progressive optic neuropathy characterized by loss of retinal ganglion cells (RGCs), resulting in functional deterioration in the visual field (VF)^1,2^. Morphological characteristics of deep optic nerve head (ONH) structures, such as lamina cribrosa (LC) and border tissue of Elschnig, are closely associated with glaucoma development and progression^3–6^. Deep ONH structural characteristics are considered to be related to the vulnerability of glaucomatous optic nerve damage^7,8^. As a marker of deformation in deep ONH structure, the location where the externally oblique border tissue (EOBT) is elongated the most was reported to coincide with the location of glaucomatous optic disc damage^8,9^. It is also co-localized with the site of LC defect or choroidal microvascular dropout^10^, suggesting that the area exposed to maximum stress in the ONH may be more susceptible to various insults that induce glaucoma.

Once glaucomatous optic disc damage occurs, RGC axons involved in this area die, resulting in a functional deficit with a visual field (VF) defect^1^. Loss of RGC axons can be detected as a thinning of the peripapillary retinal nerve fiber layer (pRNFL) and/or macular RGCs through optical coherence tomography (OCT). Although both pRNFL and macular RGCs are impaired as a result of the glaucomatous damage in ONH, the amount of thinning of the pRNFL or RGCs can differ depending on the location and extent of damage in the ONH^11,12^. In other words, the extent and location of the maximum stress induced by EOBT elongation in deep ONH may affect pRNFL and macular RGC differently. However, it has not been investigated previously whether macular RGC thickness can be affected by border tissue deformation depending on its length and location of its maximal deformation. In addition, as the thickness of macular RGCs is more directly related to the functional impairment with the center involving the VF defect^12–14^, characteristics of EOBT that reflect macular RGC loss may have useful clinical relevance.

In this study, we assessed the EOBT in the deep ONH of eyes with open-angle glaucoma (OAG) and determined the longest EOBT length and its location. We then investigated how these factors affect the macular GCIPL thickness and pRNFL thickness in eyes with OAG.

## Methods

### Subjects

This cross-sectional study recruited subjects from an ongoing retrospective cohort study which included patients who had been diagnosed with open-angle glaucoma (OAG) between 2007 and 2020 and had been followed up for at least 5 years at Samsung Medical Center (Seoul, Korea). This study was approved by the Samsung Medical Center Institutional Review Board (IRB file No. 2020–07-141–001) and adhered to the tenets of the Declaration of Helsinki. The Samsung Medical Center IRB waived the requirement for informed consent considering the retrospective nature of the study.

Patients satisfying the following criteria were included in this study: (1) patients who underwent spectral-domain (SD)-OCT examination analyzing the deep ONH structure including EOBT during the follow-up period and (2) patients who had EOBT present on SD-OCT scans of the ONH. Patients with concomitant ocular or systemic diseases that could affect VF tests, such as a history of vision-threatening retinal disease (e.g., retinal detachment, retinal vein occlusion) or neurologic disease, were excluded. To exclude the eyes with pathologic myopia from the study, those with an axial length (AL) > 28 mm or those with an AL > 26.5 mm accompanied by myopic degeneration or retinoschisis around the ONH or macula were excluded. In the case of bilateral glaucoma, one eye was randomly selected and included in the study.

OAG was diagnosed by two glaucoma specialists (J.C.H. and C.K.) according to the following criteria: (1) the presence of glaucomatous optic disc changes, (2) an open angle on gonioscopy without any findings suggesting secondary glaucoma, and (3) confirmed glaucomatous VF defects by more than one reliable test. Glaucomatous VF defect was confirmed when the two of the following criteria were satisfied: (1) a cluster of three points with a probability less than 5% on the pattern deviation map in at least one hemifield, including at least one point with a probability less than 1% or a cluster of two points with a probability less than 1%; (2) a glaucoma hemifield test result outside the normal limits; or (3) a pattern standard deviation (PSD) of 95% outside the normal limits.

All participants underwent a comprehensive ophthalmic examination, including slit-lamp biomicroscopy, Goldmann applanation tonometry, gonioscopic examination, dilated stereoscopic examination of the ONH, color and red-free fundus photography (TRC-50DX model; Topcon Medical System, Inc., Oakland, NJ, USA), automated perimetry using a central 30–2 Humphrey field analyzer (HFA model 740; Humphrey Instruments, Inc., San Leandro, CA, USA) with the Swedish interactive threshold algorithm standard, AL measurement (IOL Master®; Carl Zeiss Meditec, Jena, Germany), ultrasonographic pachymetry (Tomey SP-3000; Tomey Ltd., Nagoya, Japan), and Cirrus OCT (Carl Zeiss Meditec, Dublin, CA, USA). Systolic and diastolic blood pressure (SBP and DBP, respectively) were measured once at the initial visit.

Baseline intraocular pressure (IOP) was defined as the IOP measured at the initial visit. Mean IOP and visual field parameters (visual field index, mean deviation, and PSD) were calculated from the average of each measurement taken within 1 year of the measurement of EOBT using SD-OCT.

### Measurement of the extent and location of the EOBT

**Figure 1:**
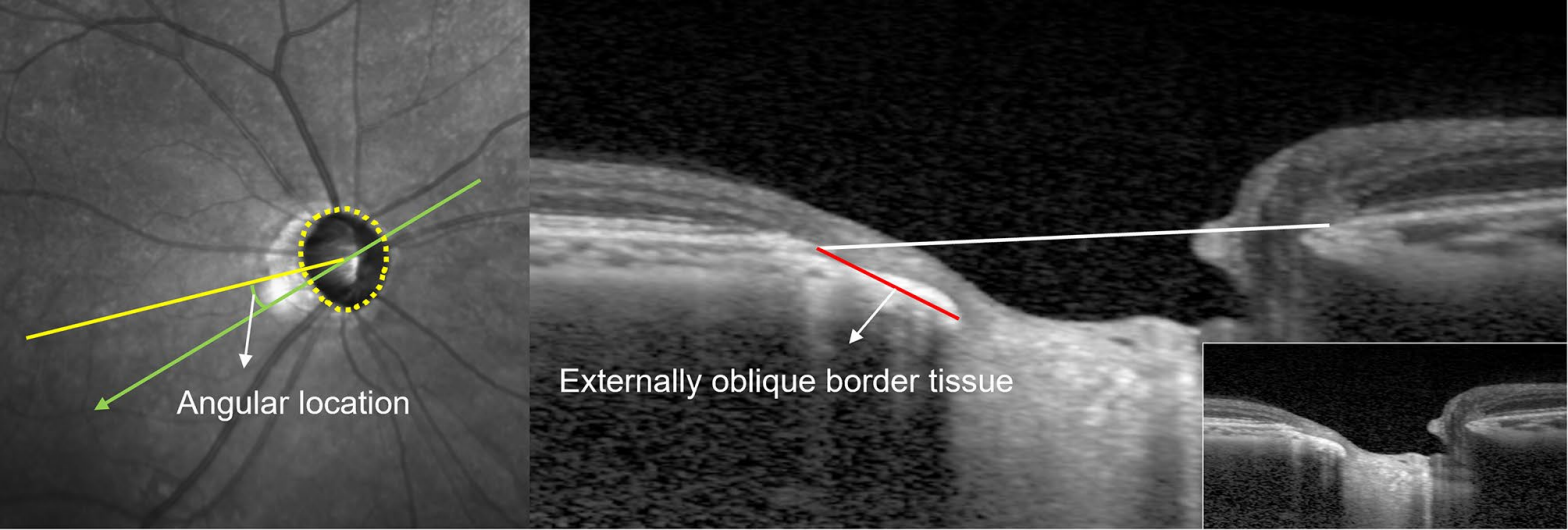
Measurement of the extent and location of the externally oblique border tissue (EOBT). The angular location of the EOBT was defined as the angle between the location of the maximum EOBT length and the FoBMO axis, the line connecting the center of the BMO and fovea (left). The extent (red line) and location of maximum length of EOBT among all scans were determined.

The maximum EOBT length and location of its maximal length were determined using enhanced depth imaging spectral-domain OCT (EDI SD-OCT; Heidelberg Engineering, Heidelberg, Germany) as previously described^8^. Briefly, OCT scans were obtained using 48 radial-line B-scans (each at an angle of 3.75°) centered on the ONH. The decision of whether the EOBT is present or absent in each case was made based on the subjective judgment of a glaucoma specialist (J.C.H.). EOBT length was defined as the length between the two endpoints of the EOBT tissue, and its maximum value among all OCT scans was used in the analysis (Fig. 1). Built-in software was used for the measurement. If the OCT image quality was insufficient to distinguish BMO or border tissue, the next scan was used. The location of EOBT was defined as the angle between the location of the maximum EOBT length and the FoBMO axis, the line connecting the center of the ONH and fovea (Fig. 1). If the angular location was below the FoBMO axis, the measurement was given a positive value; if it was above the FoBMO axis, a negative value was given. Two observers (D.Y.P. and Y.K.J.) who were blinded to the clinical information independently measured the length and location of the longest EOBT. The average values of EOBT length and location of the longest EOBT were used in the subsequent analyses.

### OCT measurement for pRNFL thickness and macular GC-IPL thickness

For the measurement of pRNFL thickness and macular GCIPL thickness, optic disc and macular scans were performed using Cirrus SD-OCT (Carl Zeiss Meditec). The pRNFL parameters collected for the analysis included average thickness and 4-quadrant thickness (superior, nasal, inferior, temporal). For the macular GCIPL parameters, the average thickness and 6 sectoral thicknesses (superotemporal, superior, superonasal, inferonasal, inferior, inferotemporal) were collected for the analysis. To investigate the relationship between the OCT parameters and EOBT measurements, Cirrus OCT exams that had been performed within 6 months of EOBT measurements were selected. OCT exams with a signal strength ≥ 7 and without motion artifacts or segmentation errors were included in the analysis.

### Statistical analysis

Interobserver (measured by D.Y.P. and Y.K.J.) reproducibility of OCT measurements was assessed by calculating the intraclass correlation coefficients (ICCs). The interobserver ICC (95% confidence interval) for the extent and location of the maximal EOBT was 0.975 (0.952–0.998) and 0.954 (0.914–0.990), respectively. Pearson’s correlation analysis was performed to evaluate the correlation between the EOBT length and macular GCIPL and pRNFL parameters. Kruskal–Wallis test and Fisher’s exact test were performed to compare the means and frequencies of the clinical variables among the four groups classified by the angular location of the longest EOBT. Univariable and multivariable regression analyses using a generalized linear model were conducted to identify factors associated with macular GCIPL thickness and pRNFL thickness. Variables with a *p*-value < 0.1 in the univariable analysis were included in the multivariable analysis. In multivariable regression analysis, two models were used to avoid multicollinearity. A *p*-value < 0.05 was considered statistically significant. Statistical analyses were performed using R statistical package version 3.6.3 (R Foundation for Statistical Computing, Vienna, Austria).

## Results

This study included a total of 149 eyes from 149 patients with OAG. The mean patient age was 59.4 ± 13.4 (standard deviation, SD) years old, and 87 eyes (58.4%) were from male patients. The baseline and mean IOP of the patients were 17.1 ± 4.2 mmHg and 14.7 ± 3.0 mmHg, respectively. The demographic and clinical characteristics of the subjects included in this study are presented in Table 1.

**Table 1:** Clinical characteristics of the subjects included in the study.

Variables	Description (n = 149)
Age, years	59.4 ± 13.4
Gender, male	87 (58.4)
SBP, mmHg	124.5 ± 13.6
DBP, mmHg	74.6 ± 11.1
Baseline IOP, mmHg	17.1 ± 4.2
Mean IOP, mmHg	14.7 ± 3.0
Central corneal thickness, μm	529.6 ± 36.4
Axial length, mm	25.2 ± 1.5
VFI	79.9 ± 17.2
MD, dB	− 7.0 ± 5.6
PSD, dB	8.7 ± 4.2
**EOBT parameters**	
Maximum EOBT length, μm	425.3 ± 286.1
Location of maximum EOBT, °	22.0 ± 37.3
Location of maximum EOBT	
EOBT location > 50°	29 (19.5)
20° < EOBT location ≤ 50°	47 (31.5)
− 20° < EOBT location ≤ 20°	54 (36.2)
− 50° < EOBT location ≤ -20°	19 (12.8)
**OCT parameters**	
RNFL, average, μm	69.4 ± 10.3
RNFL S, μm	82.5 ± 18.6
RNFL N, μm	64.1 ± 10.3
RNFL I, μm	71.8 ± 15.3
RNFL T, μm	58.6 ± 12.9
Macular GCIPL, average, μm	65.6 ± 8.3
Macular GCIPL, minimum, μm	54.5 ± 10.1
Macular GCIPL S, μm	68.3 ± 11.0
Macular GCIPL SN, μm	72.2 ± 11.3
Macular GCIPL IN, μm	67.5 ± 11.0
Macular GCIPL I, μm	60.4 ± 9.3
Macular GCIPL IT, μm	58.9 ± 9.5
Macular GCIPL ST, μm	65.8 ± 10.1

*SBP* systolic blood pressure; *DBP* diastolic blood pressure; *IOP* intraocular pressure; *VFI* visual field index; *MD* mean deviation; *PSD* pattern standard deviation; *dB* decibel; *EOBT* externally oblique border tissue; *RNFL* retinal nerve fiber layer; *S* superior; *N* nasal; *I* inferior; *T* temporal; *GCIPL* ganglion cell inner plexiform layer; *SN* superonasal; *IN* inferonasal; *IT* inferotemporal; *ST* superotemporal.

Data are presented as mean ± standard deviation or n (%).

First, to investigate the relationship between the length of EOBT and OCT parameters of pRNFL thickness and macular GCIPL thickness, we performed a correlation analysis among these parameters. The maximum extent of EOBT length was significantly correlated with the average and most of the macular GCIPL thickness. In contrast, the correlation between the maximum extent of EOBT length and pRNFL thickness was not significant (Table 2).

**Table 2:** Correlation analysis between maximum EOBT length and RNFL thickness and macular GCIPL thickness.

	Maximum EOBT length, μm	
	Correlation coefficient*	*p* value
RNFL, average, μm	0.099	0.230
RNFL S, μm	0.043	0.601
RNFL N, μm	0.110	0.202
RNFL I, μm	− 0.069	0.406
RNFL T, μm	0.110	0.167
Macular GCIPL, average, μm	− 0.200	**0.017**
Macular GCIPL, minimum, μm	− 0.083	0.316
Macular GCIPL S, μm	− 0.110	0.181
Macular GCIPL SN, μm	− 0.220	**0.008**
Macular GCIPL IN, μm	− 0.190	**0.019**
Macular GCIPL I, μm	− 0.130	0.117
Macular GCIPL IT, μm	− 0.190	**0.024**
Macular GCIPL ST, μm	− 0.160	**0.045**

*EOBT* externally oblique border tissue; *RNFL* retinal nerve fiber layer; *S* superior; *N* nasal; *I* inferior; *T* temporal; *GCIPL* ganglion cell inner plexiform layer; *SN* superonasal; *IN* inferonasal; *IT* inferotemporal; *ST* superotemporal.

*Pearson’s correlation coefficient.

Statistically significant *p*-values are shown in bold.

Next, we evaluated the pRNFL thickness and macular GCIPL thickness based on the location of the longest EOBT. We divided the patients according to the location of the longest EOBT: 29 eyes had the EOBT located at > 50°, 47 eyes showed 20° < EOBT location ≤ 50°, 54 eyes showed − 20° < EOBT location ≤ 20°, and 19 eyes showed -50° < EOBT location ≤  − 20°. The average, minimum, and sectoral macular GCIPL thicknesses were the thinnest in eyes with the EOBT located temporally (− 20° < EOBT location ≤ 20°), although statistical significance was only found for part of the sectoral macular GCIPL thickness measurements (SN and ST sectors). Eyes with the EOBT located temporally (− 20° < EOBT location ≤ 20°) were from younger individuals and had longer EOBT, longer AL, and thinner central corneal thickness. The pRNFL thickness did not significantly differ according to the location of the longest EOBT (Table 3).

**Table 3:** Comparison of clinical characteristics, RNFL thickness, and macular GCIPL thickness according to the location of maximum EOBT.

	EOBT location > 50° (n = 29)	20° < EOBT location ≤ 50° (n = 47)	− 20° < EOBT location ≤ 20° (n = 54)	− 50° < EOBT location ≤ − 20° (n = 19)	*p* value
Age, years	65.7 ± 12.5	57.6 ± 12.6	56.2 ± 13.4	63.1 ± 13.6	**0.010**
Gender, male	15 ( 51.7)	27 ( 57.4)	35 ( 64.8)	10 ( 52.6)	0.632
SBP, mmHg	122.1 ± 15.7	123.1 ± 12.4	125.5 ± 14.0	128.7 ± 11.9	0.285
DBP, mmHg	72.0 ± 12.9	73.2 ± 10.0	77.1 ± 10.4	74.5 ± 12.4	0.166
Baseline IOP, mmHg	17.6 ± 3.8	17.3 ± 5.2	17.0 ± 3.5	16.1 ± 3.4	0.564
Mean IOP, mmHg	14.2 ± 3.4	14.6 ± 3.0	15.1 ± 2.7	14.1 ± 2.8	0.442
Central corneal thickness, μm	527.3 ± 36.5	528.0 ± 32.5	524.7 ± 39.5	551.2 ± 30.9	**0.026**
Axial length, mm	24.3 ± 1.2	25.1 ± 1.3	25.9 ± 1.6	24.7 ± 1.3	** < 0.001**
VFI	80.1 ± 14.3	83.9 ± 12.1	78.7 ± 19.8	73.5 ± 22.6	0.155
MD, dB	− 7.0 ± 4.7	− 5.8 ± 4.3	− 7.3 ± 6.3	− 9.1 ± 7.1	0.222
PSD, dB	8.8 ± 4.2	8.2 ± 4.1	8.5 ± 4.4	10.0 ± 4.1	0.473
Maximum EOBT length, μm	306.6 ± 195.9	409.6 ± 250.2	574.9 ± 339.1	318.3 ± 214.4	** < 0.001**
Location of maximum EOBT, °	73.4 ± 7.1	40.0 ± 11.8	− 0.1 ± 12.9	− 38.1 ± 10.8	** < 0.001**
RNFL, average, μm	68.7 ± 12.0	69.6 ± 11.0	69.4 ± 9.7	70.3 ± 7.4	0.946
RNFL S, μm	84.3 ± 22.4	83.4 ± 20.3	81.5 ± 15.8	80.1 ± 16.5	0.846
RNFL N, μm	66.0 ± 13.1	63.8 ± 11.4	63.9 ± 8.7	62.7 ± 6.2	0.715
RNFL I, μm	66.9 ± 14.3	71.7 ± 13.9	71.9 ± 13.5	79.4 ± 21.7	0.161
RNFL T, μm	57.4 ± 16.3	59.6 ± 13.5	58.4 ± 11.5	58.8 ± 9.0	0.939
Macular GCIPL, average, μm	68.6 ± 9.3	65.9 ± 8.6	63.6 ± 7.8	65.8 ± 6.5	0.102
Macular GCIPL, minimum, μm	54.3 ± 12.9	54.8 ± 10.0	54.5 ± 9.6	54.1 ± 7.4	0.992
Macular GCIPL S, μm	70.9 ± 14.1	68.9 ± 11.8	66.4 ± 9.0	68.4 ± 7.9	0.390
Macular GCIPL SN, μm	77.2 ± 12.3	72.2 ± 11.7	69.3 ± 10.4	72.6 ± 8.6	**0.040**
Macular GCIPL IN, μm	69.9 ± 13.0	68.0 ± 11.2	65.7 ± 9.4	67.6 ± 11.6	0.428
Macular GCIPL I, μm	62.3 ± 10.9	60.4 ± 8.8	59.3 ± 8.5	60.6 ± 10.2	0.632
Macular GCIPL IT, μm	61.0 ± 10.9	58.6 ± 8.7	57.4 ± 8.5	61.1 ± 11.2	0.342
Macular GCIPL ST, μm	70.6 ± 11.6	67.1 ± 8.9	62.5 ± 9.6	64.7 ± 8.7	**0.012**

*RNFL* retinal nerve fiber layer; *GCIPL* ganglion cell inner plexiform layer; *EOBT* externally oblique border tissue; *SBP* systolic blood pressure; *DBP* diastolic blood pressure; *IOP* intraocular pressure; *VFI* visual field index; *MD* mean deviation; *PSD* pattern standard deviation; *dB* decibel; *S* superior; *N* nasal; *I* inferior; *T* temporal; *SN* superonasal; *IN* inferonasal; *IT* inferotemporal; *ST* superotemporal.

Data are presented as mean ± standard deviation or n (%).

P values were calculated from Kruskal–Wallis test or Fisher’s Exact test.

Statistically significant *p*-values are shown in bold.

Multivariable regression analysis revealed that the average macular GCIPL thickness was significantly thinner in eyes with less pRNFL thickness, eyes with longer EOBT or AL, and eyes with EOBT located more temporally (Table 4). The average pRNFL thickness was not associated with the EOBT length or location of the longest EOBT (Table 5).

**Table 4:** Univariable and multivariable regression analysis of factors associated with average macular GCIPL thickness.

	Univariable		Multivariable			
			Model 1		Model 2	
	Beta (95% CI)	*p* value	Beta (95% CI)	*p* value	Beta (95% CI)	*p* value
Age, per 1 year older	− 0.08 (− 0.18, 0.02)	0.121				
Gender, male	− 1.64 (− 4.35, 1.07)	0.237				
DBP, mmHg	0.08 (− 0.03, 0.2)	0.174				
Baseline IOP, mmHg	0.34 (− 0.09, 0.76)	0.120				
Mean IOP, mmHg	0.02 (− 0.43, 0.47)	0.929				
Central corneal thickness, μm	0.01 (− 0.03, 0.05)	0.604				
Axial length, mm	− 1.48 (− 2.34, − 0.62)	** < 0.001**				
Maximum EOBT length, μm	− 0.01 (− 0.01, 0)	**0.017**	− 0.01 (− 0.01, 0)	**0.001**	− 0.01 (− 0.01, 0)	**0.009**
Location of maximum EOBT, °	0.03 (0, 0.07)	0.082	0.03 (0, 0.06)	**0.047**		
**Location of maximum EOBT by group, reference, EOBT location > 50°**						
20° < EOBT location ≤ 50°	− 2.74 (− 6.54, 1.07)	0.161			− 2.55 (− 5.80, 0.70)	0.126
− 20° < EOBT location ≤ 20°	− 5.01 (− 8.72, − 1.3)	**0.009**			− 3.81 (− 7.14, − 0.48)	**0.027**
− 50° < EOBT location ≤ − 20°	− 2.74 (− 7.50, 2.01)	0.260			− 3.37 (− 7.4, 0.66)	0.103
RNFL, average, μm	0.40 (0.28, 0.51)	** < 0.001**	0.42 (0.31, 0.53)	** < 0.001**	0.42 (0.31, 0.53)	** < 0.001**

*GCIPL* ganglion cell inner plexiform layer; *DBP* diastolic blood pressure; *IOP* intraocular pressure; *EOBT* externally oblique border tissue; *RNFL* retinal nerve fiber layer.

Axial length and maximum EOBT length were strongly associated with each other; thus, only maximum EOBT length was included in the multivariable analysis.

Statistically significant *p*-values are shown in bold.

**Table 5:** Univariable and multivariable regression analysis of factors associated with average RNFL thickness.

	Univariable		Multivariable	
	Beta (95% CI)	*P* value	Beta (95% CI)	*p* value
Age, per 1 year older	− 0.03 (− 0.15, 0.10)	0.654		
Gender, male	− 1.84 (− 5.19, 1.51)	0.284		
DBP, mmHg	0.09 (− 0.05, 0.24)	0.217		
Baseline IOP, mmHg	− 0.47 (− 0.87, − 0.08)	**0.019**	− 0.31 (− 0.66, 0.04)	0.085
Mean IOP, mmHg	0.31 (− 0.24, 0.86)	0.268		
Central corneal thickness, μm	0.01 (− 0.04, 0.06)	0.688		
Axial length, mm	− 0.37 (− 1.47, 0.73)	0.508		
Maximum EOBT length, μm	0 (0, 0.01)	0.230		
Location of maximum EOBT, °	− 0.02 (− 0.06, 0.03)	0.478		
**Location of maximum EOBT by group, reference, EOBT location > 50°**				
20° < EOBT location ≤ 50°	0.96 (− 3.84, 5.77)	0.695		
− 20° < EOBT location ≤ 20°	0.75 (− 3.93, 5.44)	0.753		
− 50° < EOBT location ≤ − 20°	1.66 (− 4.34, 7.67)	0.589		
Macular GCIPL, average, μm	0.61 (0.43, 0.78)	** < 0.001**	0.58 (0.41, 0.76)	** < 0.001**

*RNFL* retinal nerve fiber layer; *DBP* diastolic blood pressure; *IOP* intraocular pressure; *EOBT* externally oblique border tissue; *GCIPL* ganglion cell inner plexiform layer.

Statistically significant *p*-values are shown in bold.

**Figure 2:**
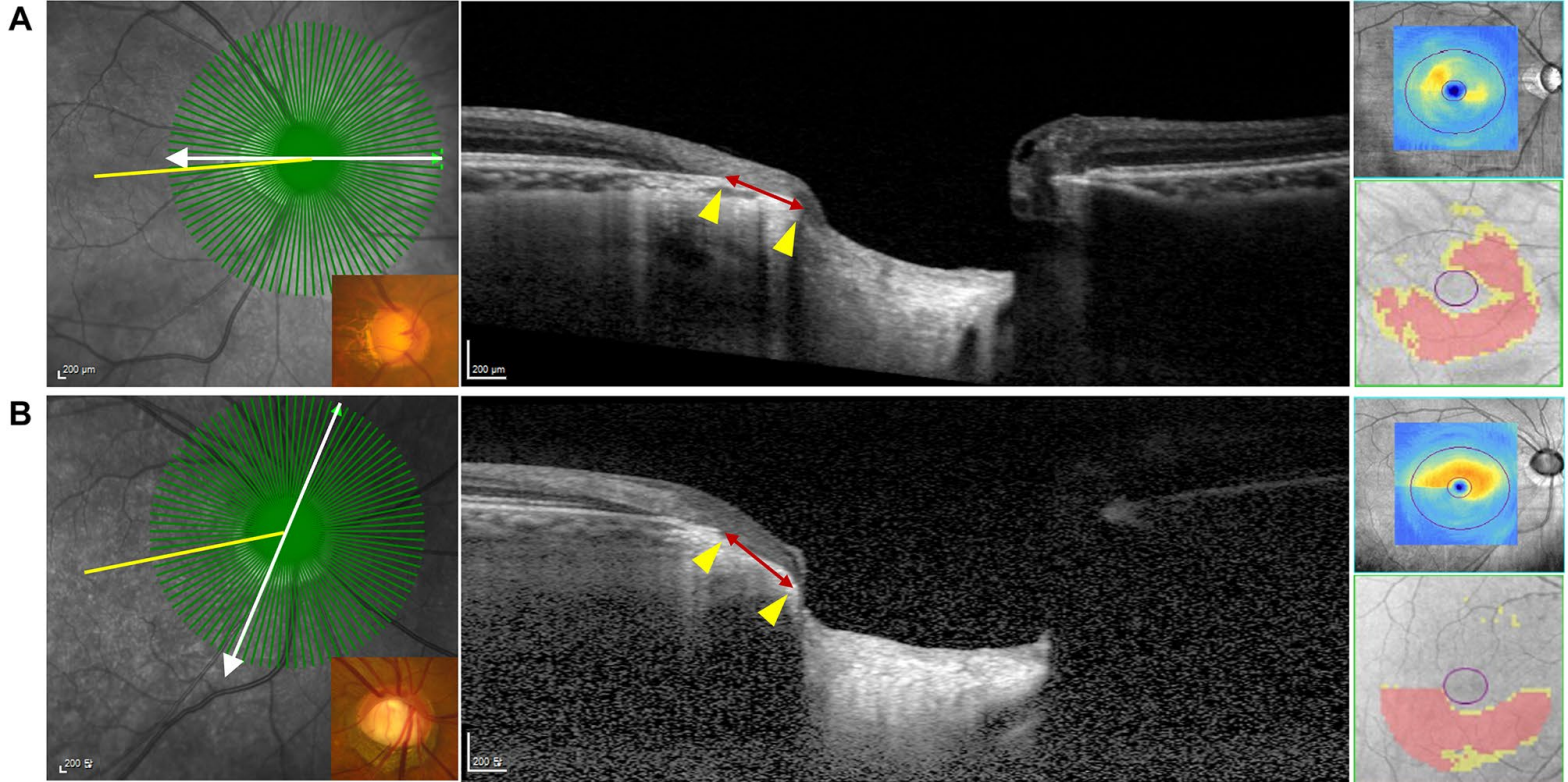
Representative cases showing the difference in the macular GCIPL thickness (right panel; upper: GCIPL thickness map, lower: GCIPL deviation map) depending on the location and length of the maximum EOBT (left panel). Yellow line: FoBMO axis; white arrow: direction of the maximum EOBT length; red line between the yellow arrowheads: maximum EOBT length. (**A**) 49-year-old male. Visual field mean deviation (VF MD) − 11.12 dB. Axial length (AL) 26.47 mm. The maximum EOBT length (513 µm) is located at 3.75° above the FoBMO axis and macular GCIPL thickness is 64 µm. (**B**) 47-year-old male. VF MD − 12.12 dB. AL 24.96 mm. The maximum EOBT length (505 µm) is located at 67.5° below the FoBMO axis and macular GCIPL thickness is 70 µm.

Figure 2 shows the representative cases of macular GCIPL thickness differences based on the location of the maximum EOBT and the maximum EOBT length.

## Discussion

In this study, we confirmed that the length of EOBT and the location of longest EOBT are closely related to the macular GCIPL thickness but not to the pRNFL thickness. Macular GCIPL thickness decreased as the length of the EOBT increased and as the most elongated EOBT was located on the temporal side of the disc closer to the fovea. In contrast, these features of EOBT did not affect the pRNFL thickness. These findings suggest that the macular GCIPL thickness, which is directly related to visual acuity and central visual field, can be affected by the morphological characteristics of deep ONH.

The border tissue of Elschnig is a fibrous tissue extending from the anterior sclera margin to the Bruch’s membrane opening^3,6^. Its externally oblique configuration is mostly observed in the inferior to temporal quadrants of the ONH^3^. Because the RGC axons pass over EOBT, the characteristics of EOBT can affect the RGC in different ways. The EOBT is positively correlated with the AL, and the inferior location of the maximum EOBT length is consistent with the location of glaucomatous damage, where the lamina cribrosa defect and choroidal microvascular dropout are frequently observed^8,10^. Our previous studies also showed that glaucomatous optic disc damage is mainly observed in the same direction of EOBT elongation in cases in which EOBT is elongated the most in the superior or inferior direction^8,9^. In this study, the multivariable regression analysis showed that the macular GCIPL thickness was associated with the EOBT length and location as well as RFNL thickness. This finding suggests that temporally elongated EOBT may be related to the thinning of macular GCIPL independent to the severity of glaucomatous damage. This may provide further evidence that EOBT may be a biomarker that is associated with the vulnerability of RGC axons to IOP-related stress and strain that induces glaucoma.

In this study, to determine the clinical significance of the temporally elongated EOBT, we divided the eyes into subgroups according to the location of the longest EOBT. Our analyses showed that eyes with the temporally elongated EOBT showed the least thickness of the macular GCIPL. A previous study reported that in cases in which the longest EOBT was located at the temporal side of the optic disc, deformation of ONH related to myopia, such as optic disc tilt and optic canal obliqueness, was the most severe^8^. In addition, temporally elongated EOBT was independently associated with the presence of normal-tension glaucoma in myopic eyes^8^. These reports are consistent with the findings of the current study, implying that the tensile strain in the temporal direction formed by the deformation of the deep ONH structure may make RCGs coming from the macular more vulnerable to damage.

EOBT is a structure closely related to the axial elongation of the globe^15^. It corresponds to gamma zone parapapillary atrophy (PPA), especially in myopic eyes^6^. Gamma zone PPA is a part of the PPA area without Bruch’s membrane and is associated with AL, whereas beta zone PPA that involves the Bruch’s membrane is related to age and glaucoma^16–18^. Therefore, the results of this study might be simply interpreted as macular GCIPL thickness decreases as EOBT increases in eyes with long AL. However, our findings suggest that the macular GCIPL thickness can be predicted based on the direction of EOBT elongation as well in an AL-independent way. In other words, macular GCIPL thinning can be accompanied by ONH deformation with temporal elongation of EOBT. In fact, even in non-myopia eyes, EOBT is frequently detected, and a prior study suggested that the direction of EOBT elongation, rather than the length of EOBT itself, aligns with the location of glaucomatous changes in ONH, increasing the susceptibility of glaucomatous damage^9^. Another study reported that gamma zone PPA in non-myopic eyes was associated with tilted discs, which may induce asymmetric strain on nerve fibers^19,20^. In addition, localized gamma zone PPA was reported to be associated with short AL and LC defects in eyes with OAG^21^. Therefore, EOBT may represent the vulnerability of glaucomatous damage rather than just reflecting the passive deformation of the ONH by axial elongation. The AL of the patient in this study was 25.2 mm; due to the relatively large number of myopic eyes included in the study, a separate analysis of non-myopic eyes was not possible. Further investigation of the effect of EOBT temporal elongation on macular GCIPL thickness in non-myopic eyes may help to better clarify the relationship between the biomechanical properties of ONH relevant to EOBT elongation and its susceptibility to glaucomatous damage.

While the characteristics of EOBT were closely related to the macular GCIPL, they were not associated with the pRNFL thickness (both average and sectoral pRNFL thickness). Two explanations are possible for this observation. First, unlike macular GCIPL, pRNFL thickness reflects the thickness of all RGC axons entering the optic disc. Due to their spatial correspondence, macular GCIPL may be more directly connected to temporally elongated EOBT in terms of structure-related strain and stress. In fact, it was an unexpected result that even the sectoral pRNFL thickness in the temporal direction was also independent of the elongation and direction of EOBT. Second, we speculate that the macular GCIPL thickness can be affected more sensitively to the damage of RGC axons derived from the macular, compared to the sectoral pRNFL thickness^13^. More research with a larger number of patients is needed to elucidate the relationship between the length and direction of EOBT and the thickness of the pRNFL in a sectoral or clockwise manner.

There are several limitations to this study. First, this study did not include normal control subjects, so whether EOBT characteristics affect the macular GCIPL in normal subjects is unclear. Nevertheless, this study comprised individuals with varying degrees of glaucoma (mean VF MD of − 7.0 dB ± 5.6), and we confirmed that the length and location of elongated EOBT affected macular GCIPL independently of the degree of glaucomatous damage. Further study will be needed to clarify how EOBT-related strain impacts macular GCIPL or macular RGC axons in healthy eyes without glaucoma-inducing insult. Second, we did not evaluate the degree of visual field defect, its center involvement, or visual acuity as parameters potentially dependent on EOBT characteristics. Although VF or visual acuity can better represent the functional significance of the relationship between EOBT and macular GCIPL, due to their large variability, inaccuracy, and non-linear scale, we investigated the macular GCIPL thickness, which can be determined more objectively and with good reproducibility. In addition, since most (72.5%) patients included in this study had central VF defects, reaching a statistically significant relationship between EOBT features and central VF defects could be difficult. Third, the OCT measurements of the GCIPL or RNFL thicknesses in this study were not automatically corrected for AL, which may have induced a magnification effect^22^. Magnification errors may have affected both macular GCIPL and RNFL thicknesses, which are negatively correlated with AL. However, our study showed that the length or location of EOBT was associated with only macular GCIPL and not RNFL, which suggests that our findings may be reliable despite the magnification errors. Finally, this study was a retrospective study with a small number of patients. Several factors may be associated with macular GCIPL other than EOBT length or locations, such as systemic factors related to the vascular instability or anatomic configuration of the macula itself, which were not addressed in this study. The effect of deep ONH deformation on RGC axons should be further refined by a prospective study with a larger number of patients including these parameters.

In conclusion, the macular RGCs were the thinnest in the eyes with a longer EOBT located in the temporal direction. Considering that the EOBT represents the deformation of deep ONH structure as a result of stress and strain applied to the RGC axons passing through it, this finding suggests that macular RGC damage may be severe in eyes with temporally elongated EOBT, potentially exacerbating the functional deficit in glaucoma.

## Data Availability

The datasets usen in the present study are available from the corresponding author upon reasonable request. The data are not publicly available due to privacy and ethical issues.
